# Optimal surgical treatment for paratesticular leiomyosarcoma: retrospective analysis of 217 reported cases

**DOI:** 10.1186/s12885-021-09122-7

**Published:** 2022-01-03

**Authors:** Rei Kamitani, Kazuhiro Matsumoto, Toshikazu Takeda, Ryuichi Mizuno, Mototsugu Oya

**Affiliations:** Department of Urology, Keio University School of Medicine, Shinanomachi 35, Shinjuku-ku, Tokyo, 160-8582 Japan

**Keywords:** Paratesticular, Leiomyosarcoma, High inguinal orchiectomy, Testis-sparing surgery, Surgical margin

## Abstract

**Background:**

Paratesticular leiomyosarcoma (LMS) is a rare tumor. Conventionally, tumor resection by high inguinal orchiectomy is performed as the preferred treatment approach for paratesticular sarcoma. On the other hand, testis-sparing surgery has recently attracted attention as a less-invasive treatment option for paratesticular sarcoma. However, the prognostic predictors and optimal treatment strategy for paratesticular LMS remain unclear because of its rarity. In this study, we systematically reviewed previously reported cases of paratesticular LMS to evaluate the prognostic factors and establish the optimal treatment strategy.

**Methods:**

A systematic search of Medline, Web of Science, Embase, and Google was performed to find articles describing localized paratesticular LMS published between 1971 and 2020 in English. The final cohort included 217 patients in 167 articles. The starting point of this study was the time of definitive surgical treatment, and the end point was the time of local recurrence (LR), distant metastasis (DM), and disease-specific mortality.

**Results:**

Patients with cutaneous LMS had a slightly better LR-free survival, DM-free survival, and disease-specific survival than those with subcutaneous LMS (*p* = 0.745, *p* = 0.033, and *p* = 0.126, respectively). Patients with higher grade tumors had a significantly higher risk of DM and disease-specific mortality (Grade 3 vs Grade 1 *p* < 0.001, and Grade 3 vs Grade 1 *p* < 0.001, respectively). In addition, those with a microscopic positive margin had a significantly higher risk of LR and DM than those with a negative margin (p < 0.001, and *p* = 0.018, respectively). Patients who underwent simple tumorectomy had a slightly higher risk of LR than those who underwent high inguinal orchiectomy (*p* = 0.067). Subgroup analysis of cutaneous LMS demonstrated that the difference in LR between simple tumorectomy and high inguinal orchiectomy was limited (*p* = 0.212). On the other hand, subgroup analysis of subcutaneous LMS revealed a significant difference in LR (*p* = 0.039).

**Conclusions:**

Our study demonstrated that subcutaneous LMS and high-grade tumors are prognostic factors for paratesticular LMS. For subcutaneous LMS, tumorectomy with high inguinal orchiectomy should be the optimal treatment strategy to achieve a negative surgical margin.

**Supplementary Information:**

The online version contains supplementary material available at 10.1186/s12885-021-09122-7.

## Background

Soft tissue sarcomas (STSs) are relatively rare tumors, accounting for 1% of all adult neoplasms [1]. Although they are often found in extremities and the retroperitoneal region, STSs in the genitourinary region are markedly rare, accounting for approximately 2% of all STSs [2]. Among genitourinary STSs, leiomyosarcoma (LMS) is one of the common histological subtypes that sometimes develops in the paratesticular region [2–4]. Possible origins of paratesticular LMS are intratesticular seminiferous tubules, the epididymis, spermatic cord, dartos layer, and scrotal skin. According to the location of origin, it is divided into 2 types: cutaneous LMS that originates from the arrector pili muscle of hair follicle or dartos muscle of genital skin, and subcutaneous LMS that originates from smooth muscle of genital organ or the vascular muscle layer of subcutaneous tissue [5, 6].

The clinical practice guidelines for STS recommend complete surgical resection including the surrounding tissue to achieve an appropriate margin status as the standard treatment [7–10]. However, the specific treatment strategy for paratesticular LMS has not been established. Conventionally, tumor resection with high inguinal orchiectomy is performed as the preferred treatment approach for paratesticular STS [11–13]. On the other hand, testis-sparing surgery has recently attracted attention as a less-invasive treatment option for paratesticular STS [14–16]. Previous case reports of paratesticular LMS demonstrated good disease control even when treated by simple tumorectomy sparing the testis [5, 6, 17, 18]. However, the prognostic factors and optimal treatment strategy for paratesticular LMS remain unclear because of its rarity.

Regarding liposarcoma, which is another of common histological subtype of genitourinary STS, we previously performed a systematic review of case reports and revealed that complete resection with high inguinal orchiectomy is beneficial [19]. Similar to paratesticular liposarcoma, almost all prior reports of paratesticular LMS are limited to a single or a few cases. Therefore, in this study, we systematically reviewed previously reported cases of paratesticular LMS to evaluate the prognostic factors and establish the optimal treatment strategy.

## Methods

**Fig. 1:**
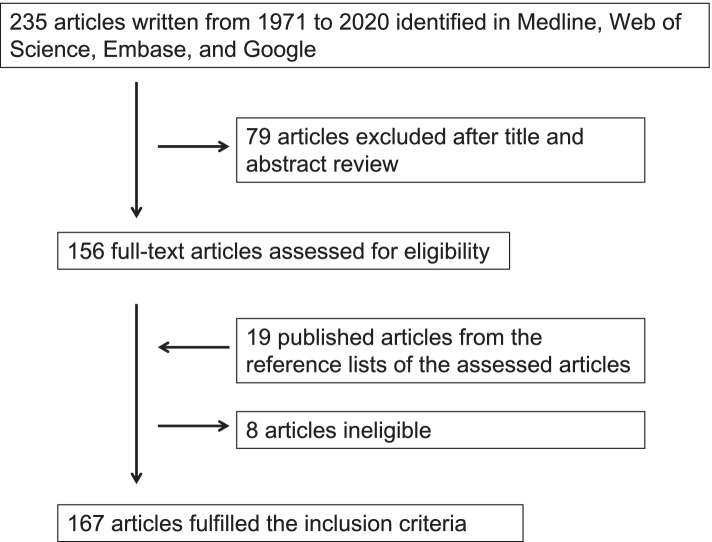
By the initial literature search, we identified and screened 235 titles and abstracts. One hundred and fifty-six articles were considered of interest and full text versions were retrieved for detailed evaluation. References cited by all 156 articles were reviewed and 19 additional articles were identified. However, 8 articles did not meet the study inclusion and were excluded. In total, 167 articles met our predefined inclusion criteria

We searched for articles describing men with paratesticular LMS published between 1971 and 2020 in English. We used Medline, Web of Science, Embase, and Google, and excluded conference proceedings or reports with only an abstract. We used the following medical subject heading terms and/or text words: ‘testicular’, ‘groin’, ‘scrotal’, ‘dartos’, ‘spermatic cord’, and ‘leiomyosarcoma’. We included only male cases of primary localized paratesticular LMS treated by surgery. Cases with no available clinicopathological data, case series describing no clinicopathological data of each individual patient, and cases mixed with the other malignancies, such as germ cell tumor, liposarcoma, and rhabdomyosarcoma, were excluded. Cases described in different reports were treated as a single case. All searches were conducted independently by two authors (RK and KM). The results were compared, and questions or discrepancies were resolved through iteration and consensus. The study flow diagram is shown in Fig. 1. In total, 167 articles fulfilled our inclusion criteria and provided a total of 217 cases for systematic review (Supplemental Table 1).

The collected clinical data included age, laterality, tumor size, tumor depth, surgical treatment, tumor grade, surgical margin, adjuvant treatment, and clinical outcomes. Regarding tumor depth, the cases were divided into 2 groups; cutaneous LMS and subcutaneous LMS [5, 6]. We regarded LMS that infiltrated subcutaneous tissue as subcutaneous LMS. Regarding tumor grade, the cases were divided into 3 groups, Grade 1 (low-grade), Grade 2 (intermediate-grade), and Grade 3 (high-grade), according to the National Federation of French Cancer Center Institute System, a scoring system based on the evaluation of the number of mitoses, percentage of necrosis, and severity of nuclear pleomorphism [20, 21].

The starting point of this study was the time of definitive surgical treatment, and the end point was the time of local recurrence (LR), distant metastasis (DM), and disease-specific mortality. Categorical variables were compared using the two-sided Fischer’s test and continuous variables were compared using the Mann-Whitney U-test. The LR-free, DM-free, and disease-specific survival (DSS) curves were constructed using the Kaplan-Meier method and compared by the log-rank test. To determine risk factors for LR and DM, multivariate analyses were performed using Cox’s proportional hazards model with stepwise forward selection. In all analyses, *P* < 0.05 was considered significant. These analyses were performed with R Statistical Language version 3.0.2.

## Results

### Patient characteristics

The characteristics of the patients are shown in Table 1. The mean age was 59.3 years (range 1–89). The mean follow-up period was 41 months.

**Table 1:** Clinicopathological characteristics

Total number of patients	217
Age (*n* = 215)	mean 59.3 years (range 1-89)
Laterality (*n* = 155)	
Left	85 (54.5%)
Right	70 (45.5%)
Depth (*n* = 208)	
Cutaneous	42 (20.2%)
Subcutaneous	166 (79.8%)
Surgical treatment (*n* = 205)	
Primary surgery	
Simple tumorectomy	71 (34.6%)
With orchiectomy	134 (65.4%)
Definitive surgery	
Simple tumorectomy	55 (26.8%)
With orchiectomy	150 (73.2%)
Adjuvant treatment (*n* = 194)Adjuvant treatment (*n* = 194)	
Adjuvant radiation therapy	31 (16.0%)
Adjuvant chemotherapy	13 (6.7%)
Tumor size (*n* = 175)	mean 6.63 cm (range 0.5-50)
Microscopic margin (*n* = 65)	
Positive	8 (12.3%)
Negative	57 (87.7%)
Tumor grade (*n* = 184)	
Grade 1	54 (29.3%)
Grade 2	63 (34.2%)
Grade 3	67 (36.4%)
Outcome (*n* = 176)	
Alive without recurrence	119 (67.6%)
Local recurrence	26 (14.8%)
Distant metastasis	45 (25.6%)
Disease-specific mortality	24 (13.6%)

### Treatment and pathological features

The characteristics of treatment and pathological features are shown in Table 1. Regarding the tumor depth, 42 (20.2%) patients had cutaneous LMS and 166 (79.8%) had subcutaneous LMS. At the timing of primary surgery, 134 patients underwent high inguinal orchiectomy. On the other hand, 71 patients underwent simple tumorectomy, and 22 subsequently required wide re-resection, including 16 with high inguinal orchiectomy. Therefore, as the definitive surgery, 150 (73.2%) patients underwent high inguinal orchiectomy and 55 (26.8%) underwent simple tumorectomy. At the timing of primary surgery, microscopic positive and negative surgical margins were observed in 16 and 49 patients, respectively. Among 16 patients with positive surgical margins, 9 patients underwent wide re-resection and 8 achieved a negative margin. Consequently, positive and negative margins after definitive surgery were observed in 8 (12.3%) and 57 (87.7%) patients, respectively. Among 194 patients with available data for adjuvant therapy, 31 (16.0%) received radiation therapy and 13 (6.7%) received chemotherapy following surgical treatment.

The clinicopathological differences between cutaneous LMS and subcutaneous LMS are shown in Table 2. Subcutaneous LMS was significantly larger than cutaneous LMS. Although those with subcutaneous LMS more often underwent definitive surgery with high inguinal orchiectomy (88.1% vs. 17.9%, *p* < 0.001), they had a significantly higher risk of a positive surgical margin (18.2% vs. 0%, *p* = 0.049).

**Table 2:** Clinicopathological differences between cases with cutaneous LMS and subcutaneous LMS

	Cutaneous LMS (*n* = 42)	Subcutaneous LMS (*n* = 166)	*p*-value
Age	63 [31-89]	62 [1- 88]	0.976
Size (cm)	4.00 [0.50-12.40]	5.00 [0.50-50.00]	0.002
Definitive surgery			
Simple tumorectomy	32 (82.1%)	19 (11.9%)	<0.001
With orchiectomy	7 (17.9%)	140 (88.1%)	
Surgical margin			
Negative	20 (100.0%)	36 (81.8%)	0.049
Positive	0 (0.0%)	8 (18.2%)	
Tumor grade			
Grade 1	13 (41.9%)	40 (27.2%)	0.101
Grade 2	11 (35.5%)	50 (34.0%)	
Grade 3	7 (22.6%)	57 (38.8%)	
Adjuvant radiation therapy			
No	34 (89.5%)	121 (81.8%)	0.333
Yes	4 (10.5%)	27 (18.2%)	
Adjuvant chemotherapy			
No	36 (94.7%)	137 (92.6%)	1.000
Yes	2 (5.3%)	11 (7.4%)	

### Survivals and prognostic factors

**Fig. 2:**
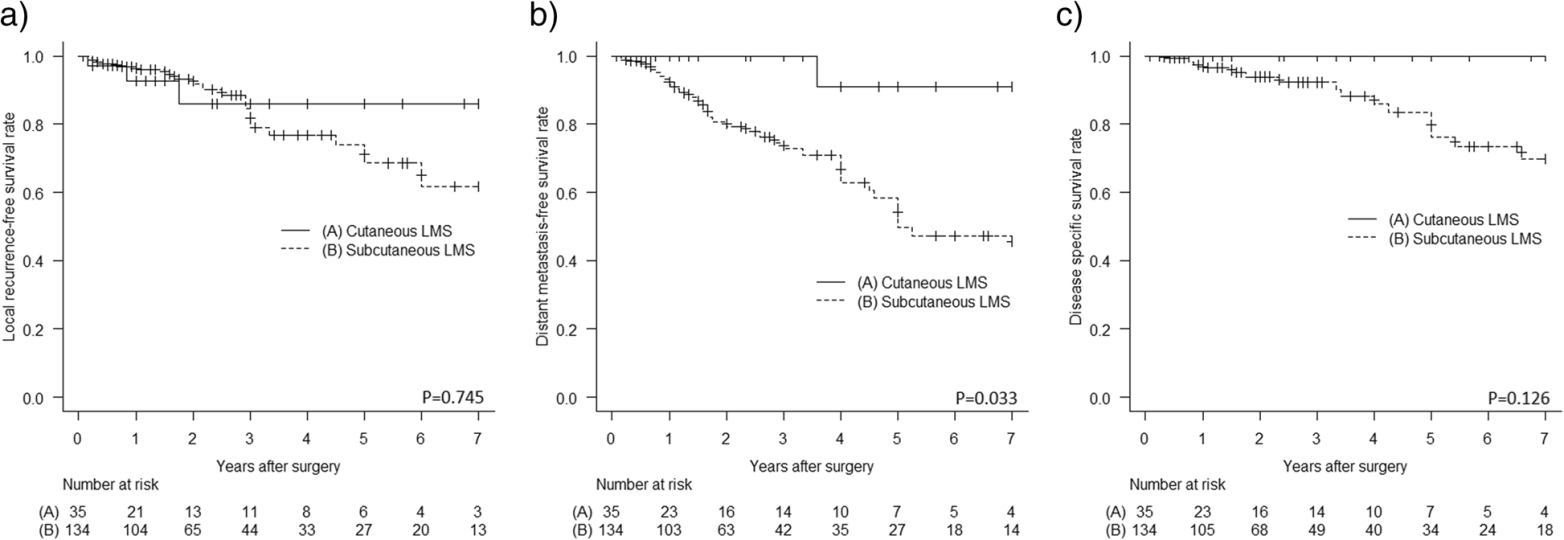
Patients with cutaneous LMS had a slightly better **a** LR-free survival, **b** DM-free survival, and **c** DSS than those with subcutaneous LMS, although the difference was not significant for LR and DSS (*p* = 0.745, *p* = 0.033, and *p* = 0.126, respectively)

**Fig. 3:**
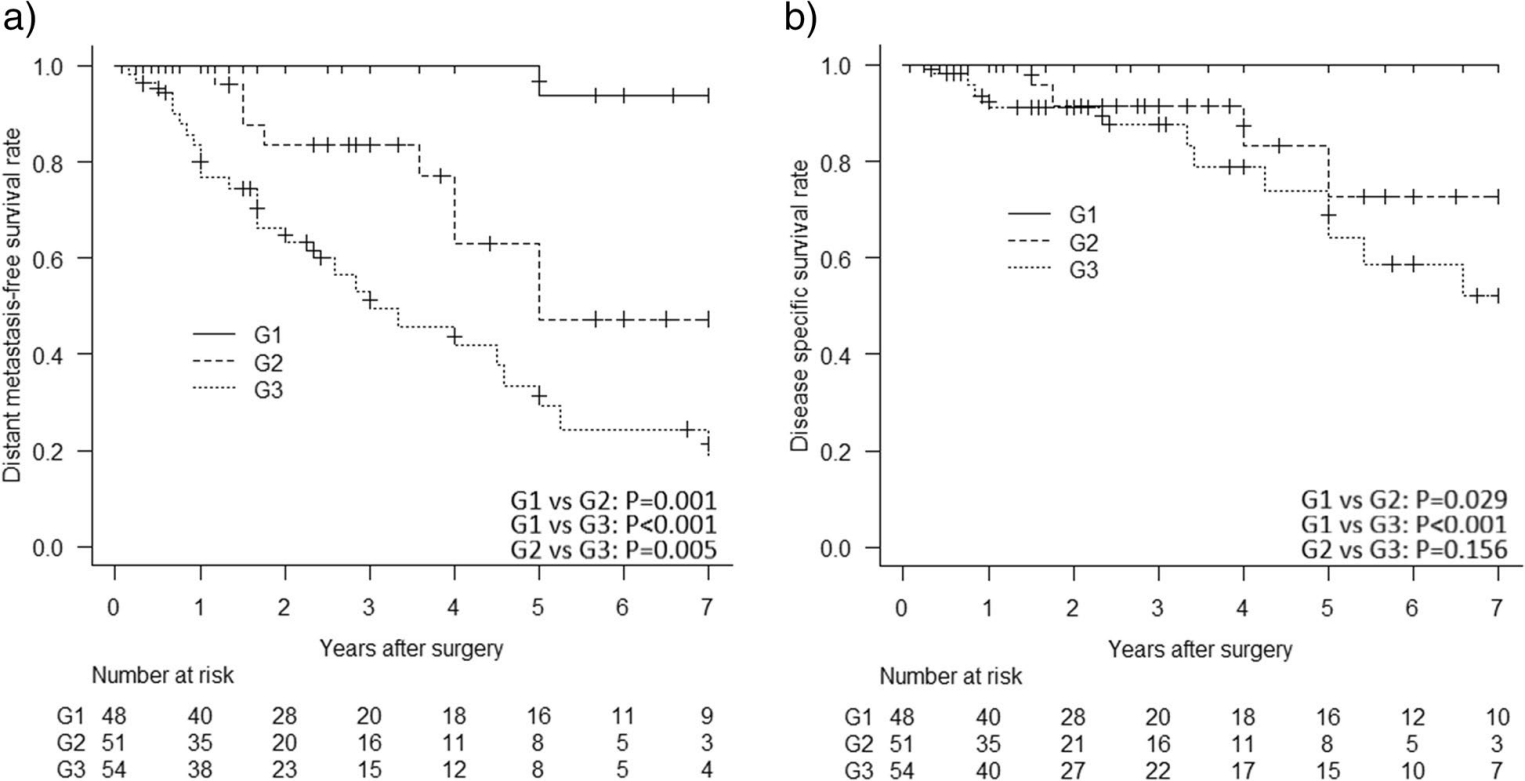
Patients with higher grade tumors had a significantly higher risk of **a** DM and **b** disease-specific mortality (Grade 3 vs Grade 1 *p* < 0.001, and Grade 3 vs Grade 1 p < 0.001, respectively)

**Fig. 4:**
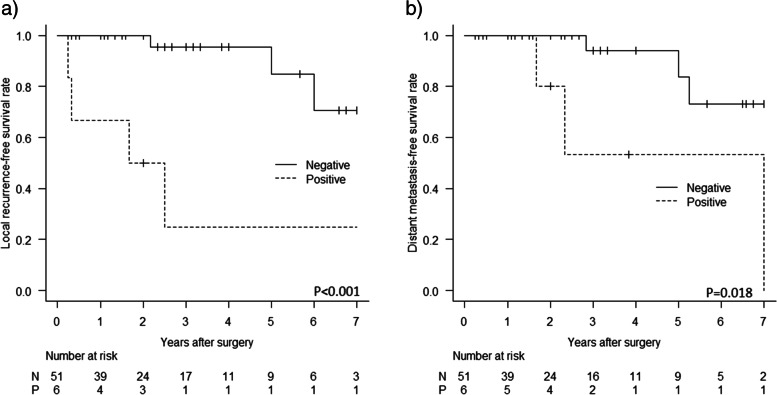
Patients with a microscopic positive margin had a significantly higher risk of **a** LR and **b** DM than those with a negative margin (p < 0.001, and *p* = 0.018, respectively)

As shown in Fig. 2a-c, Kaplan-Meier curves demonstrated that patients with cutaneous LMS had a slightly better LR-free survival, DM-free survival, and DSS than those with subcutaneous LMS, although the difference was not significant for LR and DSS (*p* = 0.745, *p* = 0.033, and *p* = 0.126, respectively). As shown in Fig. 3a-b, patients with higher grade tumors had a significantly higher risk of DM and disease-specific mortality (Grade 3 vs Grade 1 *p* < 0.001, and Grade 3 vs Grade 1 p < 0.001, respectively). In addition, as shown in Fig. 4a-b, patients with a microscopic positive margin had a significantly higher risk of LR and DM than those with a negative margin (p < 0.001, and *p* = 0.018, respectively). No significant difference in LR, DM, or DSS was observed between patients with or without adjuvant treatment.

To elucidate the risk factors for LR, we performed multivariate analyses, as shown in Table 3, which demonstrated age (years) (hazard ratio (HR) 1.09, *p* = 0.046) and microscopic positive surgical margin (HR 9.84, *p* = 0.004) to be independent risk factors. Regarding DM, subcutaneous LMS (HR 4.32, *p* = 0.047) and tumor grade (Grade 2 HR 7.06, *p* = 0.013, and Grade 3 HR 22.22, *p* < 0.001, respectively) were found to be independent risk factors. We were unable to analyze DSS due to the limited number of events.

**Table 3:** Univariate and multivariate analyses for local recurrence and distant metastasis

Characteristics	Local recurrence				Distant metastasis			
	Univariate analysis		Multivariate analysis		Univariate analysis		Multivariate analysis	
	Hazard ratio	*p*-value	Hazard ratio	*p*-value	Hazard ratio	p-value	Hazard ratio	*p*-value
Age (years)	1.04 (1.01-1.07)	0.007	1.09 (1.01-1.19)	0.046	1.01 (0.99-1.02)	0.647		
Laterality								
Left	Reference				Reference			
Right	1.12 (0.46-2.70)	0.810			0.61 (0.27-1.40)	0.242		
Tumor size (cm)	0.98 (0.88-1.09)	0.703			1.05 (1.01-1.10)	0.024		
Tumor depth								
Cutaneous	Reference				Reference		Reference	
Subcutaneous	1.19 (0.41-3.47)	0.747			3.30 (1.02-10.68)	0.047	4.32 (1.02-18.22)	0.047
Treatment								
Simple tumorectomy	Reference				Reference			
With orchiectomy	0.49 (0.22-1.07)	0.074			1.43 (0.70-2.94)	0.330		
Adjuvant radiation								
No	Reference				Reference			
Yes	1.58 (0.62-3.92)	0.346			1.32 (0.64-2.70)	0.455		
Adjuvant chemotherapy								
No	Reference				Reference			
Yes	0.67 (0.09-4.97)	0.695			1.60 (0.57-4.51)	0.373		
Microscopic margin								
Negative	Reference		Reference		Reference			
Positive	11.14 (2.47-50.20)	0.002	9.84 (2.06-46.93)	0.004	5.96 (1.14-31.05)	0.034		
Tumor grade								
Grade 1	Reference				Reference			
Grade 2	1.73 (0.52-5.72)	0.369			7.55 (1.66-35.52)	0.010	7.06 (1.50-33.19)	0.013
Grade 3	2.60 (0.91-7.40)	0.073			21.18 (4.97-90.39)	<0.001	22.22 (5.18-95.26)	<0.001

### Surgical approach and prognosis

**Fig. 5:**
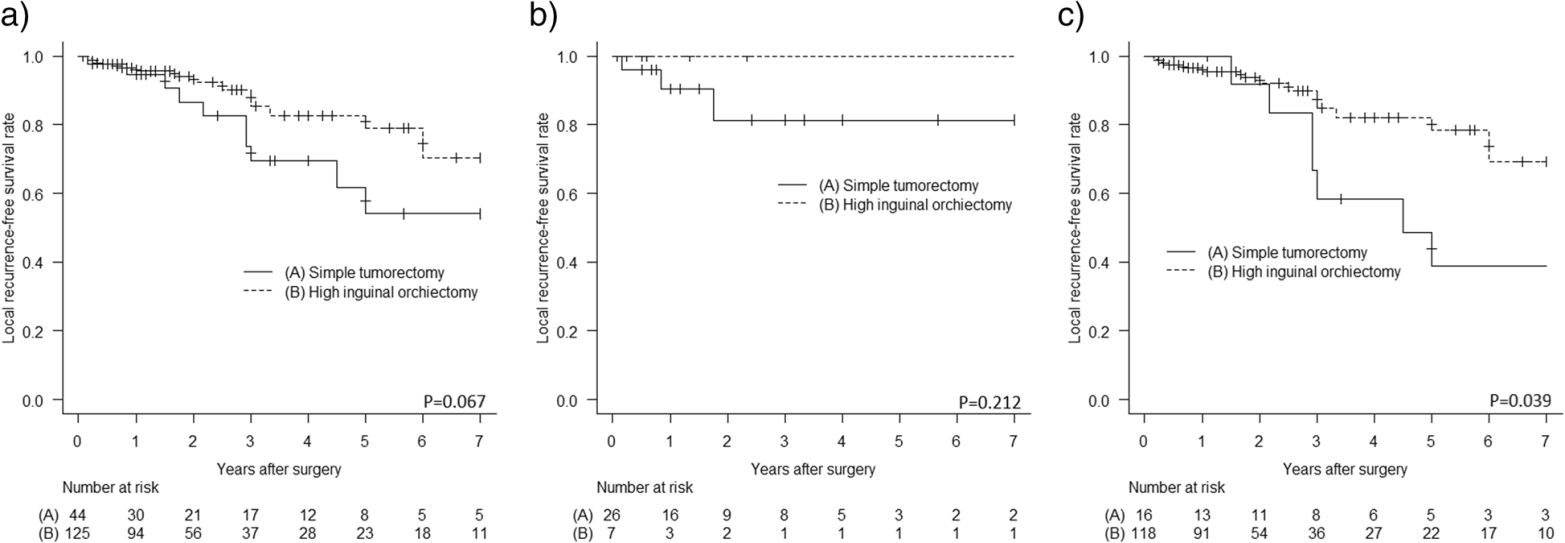
**a** Patients who underwent simple tumorectomy had a slightly higher risk of LR than those who underwent high inguinal orchiectomy, although there was no significant difference (*p* = 0.067). **b** Subgroup analysis of cutaneous LMS demonstrated that the difference in LR between simple tumorectomy and high inguinal orchiectomy was limited (*p* = 0.212). **c** Subgroup analysis of subcutaneous LMS revealed a significant difference in LR (*p* = 0.039)

Patients who underwent simple tumorectomy had a slightly higher risk of LR than those who underwent high inguinal orchiectomy, as shown in Fig. 5a, although there was no significant difference (*p* = 0.067). Subgroup analysis of cutaneous LMS demonstrated that the difference in LR between simple tumorectomy and high inguinal orchiectomy was limited, as shown in Fig. 5b (*p* = 0.212). In contrast, as shown in Fig. 5c, subgroup analysis of subcutaneous LMS revealed a significant difference in LR. (*p* = 0.039). At the primary surgery for subcutaneous LMS, patients treated by simple tumorectomy had significantly smaller tumors than those treated by high inguinal orchiectomy (4.0 cm vs 6.0 cm, *p* = 0.004), but they had a significantly higher risk of a positive surgical margin (9 of 17 vs. 5 of 27, *p* = 0.024). Among patients with a positive margin on simple tumorectomy for subcutaneous LMS, 6 underwent wide re-resection with high inguinal orchiectomy, and achieved a negative margin and subsequent good disease control.

Regarding the DM and DSS, there was no significant difference between simple tumorectomy and high inguinal orchiectomy, and the results were same even after subgroup analyses.

## Discussion

To the best of our knowledge, this is the first and largest retrospective study focusing on paratesticular LMS. We revealed that subcutaneous LMS and high grade tumors have a poorer prognosis than their counterparts. Several retrospective studies focused on LMS of the skin revealed that subcutaneous LMS had a higher risk of DM and mortality than cutaneous LMS [22–24]. Moreover, several previous studies demonstrated that tumor grade was one of the strong prognostic factors for all-site LMS [25] and genitourinary STS [2–4]. These studies were consistent with our results for paratesticular LMS.

Conventionally, complete tumor resection with high inguinal orchiectomy is recommended as the surgical treatment for paratesticular STS [11–13]. Our study suggested that patients with a positive surgical margin had a poorer prognosis than those with a negative surgical margin, being consistent with several previous studies demonstrating that the surgical margin status is associated with the recurrence and mortality of STS [25–27]. Therefore, complete tumor resection with high inguinal orchiectomy to achieve a negative surgical margin status should be recommended for paratesticular LMS. Our subgroup analysis on subcutaneous LMS demonstrated that high inguinal orchiectomy was significantly beneficial for negative margin status and subsequent local disease control, and it should be the optimal treatment strategy. Murray et al. reported that wide re-resection with negative margins improved recurrence-free and DSS rates of spermatic cord sarcoma [28]. Supporting their findings, our 6 patients with subcutaneous LMS, which exhibited positive surgical margins when treated by simple tumorectomy as the primary treatment, developed no recurrence after undergoing secondary wide complete resection with high inguinal orchiectomy. Therefore, when the appropriate margin status cannot be achieved by primary surgery, wide re-resection must be considered.

The results of our subgroup analysis of cutaneous LMS are controversial. As the majority of patients treated by unilateral radical orchiectomy had a reduced postoperative testosterone level, testis-sparing surgery has attracted attention from the perspective of ethical, cosmetic, and hormonal issues [14–16]. Paratesticular tumors can be easily evaluated using imaging tests such as scrotal ultrasonography and MRI [13, 16, 29]. Thus, testis-sparing surgery may be a treatment option when a negative surgical margin status can be guaranteed. The preoperative physical examination and imaging tests may aid in avoiding unnecessary orchiectomy, but further studies are required to confirm our results.

The current study has several limitations, which are mainly related to its retrospective nature and the large disparity among reporting styles of institutions. There may be a publication bias that led to the over-reporting of better therapeutic outcomes or conversely poorer biological outcomes of LMS. The majority of authors reported their cases within 5 years from the time of surgery and the follow-up term was insufficient for the evaluation of DSS. Some available data for the pathological and clinical status were lacking, and we were unable to provide a definitive conclusion. Regarding uterine LMS and endometrial stromal sarcoma, the expression of estrogen receptor (ER) and progesterone receptor (PR) was frequently observed in previous studies using immunohistochemistry. In addition, cases with ER/PR expression had a good response to aromatase inhibitors [30, 31]. On the other hand, LMS cases exhibiting ER/PR expression in males are markedly rare. In our study cohort, ER/PR expression was examined in only one case [32]. Therefore, its efficacy as a biomarker in paratesticular LMS remains unclear.

## Conclusions

Our study demonstrated that subcutaneous LMS and high-grade tumors are prognostic factors for paratesticular LMS. For subcutaneous LMS, tumorectomy with high inguinal orchiectomy should be the optimal treatment strategy to achieve a negative surgical margin. Re-resection in patients with positive margins may improve their prognosis.

## Supplementary Information

**Additional file 1.**

## Data Availability

All previous reports included in this study are listed in the Supplemental Table 1.

## Abbreviations

STS: soft tissue sarcomas
LMS: leiomyosarcoma
LR: local recurrence
DM: distant metastasis
DSS: disease-specific survival
HR: hazard ratio
